# The Effects of Sourdough Fermentation on the Biochemical Properties, Aroma Profile and Leavening Capacity of Carob Flour

**DOI:** 10.3390/foods14101677

**Published:** 2025-05-09

**Authors:** Gemma Sanmartín, Jose A. Prieto, Miguel Morard, Francisco Estruch, Josep Blasco-García, Francisca Randez-Gil

**Affiliations:** 1 Department of Biotechnology, Instituto de Agroquímica y Tecnología de los Alimentos, Consejo Superior de Investigaciones Científicas, Avda. Agustín Escardino, 7, 46980 Paterna, Valencia, Spain; gemma.sanmartin@iata.csic.es (G.S.); prieto@iata.csic.es (J.A.P.); miguel.morard@iata.csic.es (M.M.); josepblasco@iata.csic.es (J.B.-G.); 2 Department of Biochemistry and Molecular Biology, Universitat de València, Dr. Moliner 50, 46100 Burjassot, Valencia, Spain; francisco.estruch@uv.es

**Keywords:** baking, bread, roasted carob pods, starters, lactic acid, gassing power, VOCs

## Abstract

Roasted carob flour is a sustainable ingredient rich in dietary fiber, polyphenols, and pinitol, offering potential for both food and pharmaceutical applications. However, its high sugar content and the presence of undesirable compounds such as furans present challenges for its use in bread making. This study evaluated the effects of prolonged sourdough fermentation on roasted carob flour, with a focus on microbial dynamics and its functional and technological properties. Carob and carob–wheat sourdoughs were prepared using a mixed starter culture comprising three lactic acid bacteria (*Lactiplantibacillus plantarum*, *Fructilactobacillus sanfranciscensis*, and *Lactobacillus helveticus*) and three yeast species (*Saccharomyces cerevisiae*, *Kazachstania humilis*, and *Torulaspora delbrueckii*). The sourdoughs underwent six consecutive refreshment cycles and were analyzed to determine their pH, microbial and biochemical composition, gassing power, and volatile organic compounds (VOCs). The carob–wheat sourdough exhibited faster acidification and higher lactic acid bacteria (LAB) activity, resulting in a 90–98% reduction in the sugar content, compared to 60% in the carob sourdough. Microbial sequencing revealed that *L. plantarum* was the dominant species in all samples, while *K. humilis* and *S. cerevisiae* were enriched in carob and carob–wheat sourdough, respectively. Both types of sourdough demonstrated effective leavening in bread dough without the addition of commercial yeast. Fermentation also modified the VOC profiles, increasing esters and alcohols while reducing acids, aldehydes, ketones, and furans. While the antioxidant activity showed a slight decline, the pinitol content remained unchanged. These findings suggest that extended sourdough fermentation, supported by multiple refreshments, enhances the baking suitability of roasted carob flour and supports its application as a functional, sustainable ingredient.

## 1. Introduction

Traditionally grown in arid and semi-arid regions, the carob tree (*Ceratonia siliqua*) has been a vital component of Mediterranean agriculture for generations. Its resilience to drought, poor soils, and harsh climatic conditions makes it an essential crop in the fight against desertification. However, its cultivation has experienced a drastic decline since the early 20th century. For instance, in 1930, Spain, the world’s largest carob producer (accounting for 30% of the global output), produced 546,000 tons of carob from an area of approximately 187,000 hectares. Today, those figures have dwindled to around 40,000 hectares and 50,000 tons [1]. Nevertheless, with the rising global demand for sustainable and plant-based products, the prospects for carob cultivation appear promising, though strategies to enhance its economic and environmental value remain essential.

In recent years, there has been a renewed interest in carob trees due to the growing applications of carob pods, the pulp of their fruit. Traditionally associated with animal feed, roasted carob flour—the most common form of carob pod consumption—has emerged as a valuable ingredient in the food and pharmaceutical industries due to its healthy nutritional properties [2,3]. It is naturally sweet and gluten- and caffeine-free, making it suitable for people with celiac disease [4]. It is rich in dietary fiber, polyphenols, particularly tannins [5], and healthy inositols, mainly pinitol [6], which has antioxidant properties and helps regulate blood glucose [7,8]. Additionally, carob is believed to have several pharmacological activities, including anti-inflammatory, anti-cancer, and hypolipidemic effects [9]. Finally, locust bean gum (E-410), extracted from carob seeds, is a key stabilizer and thickener in dairy, bakery, and gluten-free products and a hydrocolloid with different industrial applications [10].

In light of these benefits, various food formulations featuring carob flour have been developed, including fiber-rich snacks, jellies, and energy bars, among others [11,12]. In confectionery products, roasted carob flour has been successfully employed as a healthier substitute for cocoa due to its similar sensorial, chemical, and biological properties, lack of stimulating alkaloids, and low fat content [13].

Bread formulations incorporating carob flour blended with wheat in varying proportions have been evaluated for their nutritional and technological characteristics. In general, the baked goods produced are associated with potential positive impacts on human health, primarily due to their higher content of fiber and antioxidants [14]. However, breads containing carob flour exhibit impaired rheological properties [14,15,16], a sweet taste, chocolate-like flavor, and a browner color compared to traditional wheat bread [17]. Additionally, the use of carob flour in baking faces several challenges, including its high sugar content, which may be seen as a drawback by both consumers and manufacturers, especially with the increasing concern over obesity and other diet-related issues [18]; the formation of undesirable compounds such as furans and methylfurans during roasting, which could pose a risk to public health [18]; and the need to develop strategies for reducing the caloric contribution of carob flour in baked products while preserving its nutritional properties.

Sourdough fermentation has the potential to enhance the nutritional and functional properties of carob flour. In artisanal bread production, sourdough—a mixture of wheat flour and water that is spontaneously fermented by endogenous microorganisms—is used as a leavening agent [19,20,21]. The propagation of the initial dough over an extended period through daily refreshments, or backslopping cycles, allows for the selection of well-adapted microorganisms, primarily lactic acid bacteria (LAB) and yeasts, which establish a mature sourdough that can be maintained for decades [22]. Alternatively, initiating a sourdough with a starter culture, followed by backslopping, can accelerate the process and facilitate the development of a specific microbial composition with an enhanced acidification capacity or flavor characteristics [19,20]. Regardless of the fermentation approach, the microbial activity improves bread’s flavor and texture [21] and offers potential health benefits [23], including reduced levels of phytates and gluten, improved digestibility, and a lower glycemic index [24,25].

Similarly, the fermentation of various legume flours has been shown to improve their digestibility and bioavailability by partially or completely eliminating anti-nutritional factors such as α-galactosides, tannins, phytic acid, and trypsin inhibitors, among others [26]. The use of sourdough fermentation with mixtures of wheat and legume flours has been reported to enhance the nutritional, textural, and sensory properties of white bread [27,28,29]. In this context, fermenting carob flour with *Levilactobacillus brevis* or *Limosilactobacillus fermentum*, in combination with *Saccharomyces cerevisiae*, for 24 h at 30 °C reduced the crumbliness and enriched wheat bread with dietary fiber and phenolic antioxidants [30]. However, the reduction in the sugar content was minimal, and the microbial counts remained relatively low.

The fermentation of carob flour combined with proso millet flour and bran by *Limosilactobacillus fermentum* and *Kluyveromyces marxianus* at 35 °C for 16 h has also been evaluated for use in creating a functional ingredient in the preparation of gluten-free flatbread [31]. The microbial starter was effective in reducing the sugar content of the flour blend and improving the acidity and rheological properties of the gluten-free bread. Nevertheless, the yeast counts remained low, and the leavening of the gluten-free dough still depended on the use of commercial baker’s yeast [31]. This was likely due to the short fermentation time and the absence of successive refreshments, which are necessary to produce mature sourdoughs with high cell densities and metabolic activity [21]. In this regard, it is worth noting that the regulations and codes of practice in many European countries stipulate that bread elaborated with sourdough must not contain more than 0.2% (based on the flour weight) commercial baker’s yeast [32]. Thus, the potential of carob sourdough as a leavening and aroma-enhancing agent in baking remains to be fully determined.

In this study, we conducted the first analysis of the microbial dynamics in sourdoughs based on carob and carob–wheat blends, using defined starter cultures composed of various lactic acid bacteria and yeast species. The baking-relevant attributes and key nutritional parameters of carob flour were monitored over the multiple backslopping cycles required to establish a mature sourdough ecosystem. Our findings identify the microbial species that successfully colonize carob-based sourdoughs and elucidate how successive refreshments influence their technological and nutritional characteristics. Prolonged fermentation reduced the inherent limitations of carob flour and enhanced its functionality in baking applications. These insights may support the broader use of carob flour, aligning with consumer interest in sourdough products and healthier baked goods.

## 2. Materials and Methods

### 2.1. Strains, Media, and Culture Conditions

The yeast strains *Saccharomyces cerevisiae* G40, *Kazachstania humilis* G50, and *Torulaspora delbrueckii* G118 and lactic acid bacteria (LAB) *Lactiplantibacillus plantarum* (formerly *Lactobacillus plantarum*) G103, *Fructilactobacillus sanfranciscensis* G225, and *Lactobacillus helveticus* G229, all of them isolated from sourdough in our laboratory [33], were used throughout this work.

Previously described standard methods were followed for media preparation [34]. For sourdough inoculation, yeast cells were propagated in liquid (2% glucose, 2% peptone, 1% yeast extract) YPD at 30 °C for 24 h under agitation (180 rpm). LAB were cultivated in liquid Sourdough Medium, SDM (DSMZ-GmbH, medium 225), at 30 °C for 72 h and under static conditions. Cells were harvested by centrifugation (3220× *g*, 5 min, 4 °C), washed twice in sterile 0.9% NaCl, and re-suspended in tap water to a final cell concentration of a DO_600_ of ~2.0 (approx. 2 × 10^7^ cells/mL).

### 2.2. Sourdough Preparation

The initial sourdough was prepared by hand-mixing 50 g of carob flour (C) or a 50:50 (*w*:*w*) mixture of carob–wheat flour (CW) with 75 mL of tap water, resulting in a dough yield (DY) of 250. CAROCHOC-3 (G.A. TORRES, S.L., Turis, Valencia, Spain), a roasted and milled carob flour containing 33.6% sugars, 18.4% fiber, 4.8% protein, 3.3% ash, and 5% moisture, and Florencia wheat flour (Harinas Polo, Villanueva de Gállego, Zaragoza, Spain), a high-strength flour (400–440 W, 0.90 P/L, 33% gluten) suitable for long fermentation processes, were used. The inoculum consisted of a microbial consortium comprising 0.5 OD_600_ units of yeast pre-culture and 0.25 OD_600_ units of bacterial pre-culture, corresponding to approximately 5.1 log_10_ colony-forming units (cfu) of yeasts and 4.8 log_10_ cfu of lactobacilli per gram of sourdough (fresh basis—f.b.). Fermentation was carried out in a closed container for 24 h at 25 °C under static conditions, followed by six backslopping steps (R1_24 to R6_24) under the same parameters. In the final step, fermentation was extended to 48 h (R6_48). Each refreshment step involved mixing 50 g of the previously fermented dough with 50 g of flour and 75 mL of tap water. Throughout the process, the fermentation progress was monitored by measuring the dough pH after each backslopping cycle. Samples of the initial unfermented sourdough (C0 and CW0) and the corresponding fermented sourdoughs (CR3_24, CWR3_24, CR6_24, CWR6_24, CR6_48, and CWR6_48) were collected and frozen at −80 °C for further assays or processed immediately for total titratable acidity (TTA) and microbiological analysis. Three independent replicates of each sourdough type (C and CW) were prepared and analyzed in this study.

### 2.3. Sourdough Homogenization, pH, and TTA Determination

pH values were measured with a portable pH Meter 98190 (HANNA Instruments, Eibar, Spain) equipped with a food penetration probe. An AQUALAB 4TE water activity meter instrument (Addium Inc., Pullman, WA, USA) was used to measure the water activity of the sourdough. Sourdough homogenates were prepared by blending 10 g of sourdough with sterile 0.9% NaCl to a final volume of 100 mL, followed by homogenization for 4 min using a BagMixer 400 blender (Interscience, Saint-Nom-la-Bretêche, France). For TTA analysis, the homogenate was centrifuged (3220× *g*, 15 min, 4 °C), and 25 mL of the supernatant (analyzed in triplicate) was titrated with an automatic potentiometric titrator Excellence T5 (Mettler Toledo, Columbus, OH, USA). The results were expressed as the volume (ml) of 0.1 N NaOH needed to reach a pH of 8.5.

### 2.4. LAB and Yeast Counting

To quantify the yeast present in each sourdough, 10-fold serial dilutions of the initial homogenate were prepared, and 100 μL of each dilution was plated in triplicate on a YPD agar medium supplemented with 100 μg/mL kanamycin. The plates were incubated at 30 °C for 48 h, after which the colonies were manually enumerated. Only dilutions yielding 30–300 colonies per plate were considered for analysis.

LAB enumeration was performed similarly, except that cells were grown under microaerophilic conditions (BD GasPak™ EZ Campy, Fisher Scientific, Pittsburgh, PA, USA) at 30 °C for 72 h on SDM agar supplemented with 0.1 g/L cycloheximide. In all cases, the yeast and LAB abundances were expressed as the mean (±SD) of the log_10_-transformed CFU per gram of sourdough (f.b.).

### 2.5. Sequencing

The amplicon sequencing protocol targeted the V3–V4 regions (459 bp) of the 16S rRNA gene and the ITS2 region of fungal nuclear ribosomal DNA (rDNA), using primers designed around conserved regions. For ITS2, the primers ITS3-F_KYO2 (18S SSU 2029–2046) and ITS4_KYO1 (5.8S 2390–2409) were used [35].

Following the Illumina amplicon library preparation protocol, DNA amplicon libraries were generated using a limited-cycle PCR with the KAPA HiFi HotStart ReadyMix PCR Kit (cat# KK2602, Roche Molecular Systems, Inc., Santa Clara, CA, USA). For ITS2, the PCR conditions included an initial denaturation at 95 °C for 3 min, followed by 28 cycles of amplification (95 °C for 30 s, 58 °C for 30 s, 72 °C for 30 s) and a final extension at 72 °C for 5 min. Illumina sequencing adaptors and dual-index barcodes were then added to the amplicons by using the Nextera XT index kit v2 (cat# FC-131-2001, Illumina, San Diego, CA, USA) in a second PCR. Libraries were normalized and pooled before sequencing.

The sample containing indexed amplicons was loaded onto a NextSeq 2000 P1 reagent cartridge (cat# 20075294, Illumina, San Diego, CA, USA) and onto the instrument, along with a flow cell. Automated cluster generation and paired-end sequencing with dual-index reads were performed using a 2 × 300 bp run. All reads were trimmed and quality-checked using fastp [36] with a minimum read length of 50 bp, a minimum trimming quality mean of 20, and a window size of 100.

### 2.6. Bacterial and Fungal Analysis

The bacterial data were analyzed using QIIME 2 (version 2024.5.0) [37]. Reads were first denoised using the QIIME 2 plugin DADA2 (q2-dada2). The resulting amplicon sequence variants (ASVs) were then phylogenetically classified using the scikit-learn classifier plugin in QIIME 2 [38], trained on the SILVA 138-99-nb database. Mitochondrial and chloroplast sequences were removed, and a contingency filter was applied, retaining only sequences present in at least two samples with a minimum frequency of 15.

ASVs assigned to the genus *Lactiplantibacillus* were manually analyzed in NCBI and aligned with MAFFT to the Sanger sequence obtained from a single culture of the inoculated bacterium to determine the species-level classification. As the abundance of reads assigned to other bacterial taxa was very low, only ASVs corresponding to the genus *Lactiplantibacillus* were represented, all of which were manually verified and classified as one of the inoculated bacteria.

The fungal data were first preprocessed using ITSxpress (version 2.1.1) [39] to extract the ITS2 region from the reads. The resulting data were then denoised using the DADA2 plugin in QIIME 2, as previously described, and the resulting ASVs were phylogenetically classified using the scikit-learn classifier trained on the UNITE fungal database (version 10.0, released on 04.04.2024) [40]. Due to the high diversity, the filtering criteria were based on the sequence frequency, setting a minimum threshold of 100 per ASV and ensuring the presence of each ASV in at least three samples.

### 2.7. Metabolite Target and Sugar Analyses

The determination of the main fermentation metabolites, glycerol, ethanol, and lactic and acetic acid, was carried out by High-Performance Liquid Chromatography (HPLC), using a chromatograph (Waters, Milford, MA, USA) equipped with a quaternary pump, micro vacuum degasser, and refraction index and variable wavelength (PDA) detectors. Samples of the sourdough homogenates were centrifuged (10,000× *g*, 15 min, 4 °C) and filtered through 0.45 μm hydrophilic PTFE filters, and 20 μL was injected into an Ion Exclusion 6 μm column (8 × 300 mm; SH-1011, Shodex, Tokyo, Japan) with a guard column SH-G. Chromatographic separation was conducted under isocratic conditions with a 0.5 mL/min flow rate of 1.5 mM H_2_SO_4_ at 50 °C. The run time, including separation and column washing, was 40 min.

High-performance anion exchange chromatography (HPAEC) analyses of sourdough sugars were carried out using an ICS-6000 series Dionex liquid chromatograph (Sunnyvale, CA, USA) equipped with an AS-AP autosampler with a 25 µL loop, a CarboPac PA1 column (4 × 250 mm) connected to the corresponding guard column CarboPac PA1 (4 × 50 mm), and a pulsed amperometric detector (HPAEC-PAD). The mobile phase, maintained under a constant helium atmosphere, consisted of (A) Milli-Q water, (B) 0.5 M NaOH, and (C) 1 M sodium acetate. The following elution program was used: at 0–11 min, 20% B under isocratic conditions; at 11–30 min, a gradient from 20% B to 20% B + 5% C; and at 30–40 min, 20% B + 5% C under isocratic conditions, followed by column washing and re-equilibration, with a total run time of 50 min. The flow rate was 1.0 mL/min, and the injection volume of clarified and filtered sourdough homogenates was 25 µL. Chromatographic data were recorded using Chromeleon™ Chromatography Data System (CDS) software (Thermo Fisher Scientific, Waltham, MA, USA).

In all cases, quantification was performed using external standards for each compound. Calibration curves were constructed, in duplicate, with at least six points (R^2^ > 0.99) in the range of the analyzed concentrations. The values are expressed as mg per g of sourdough (f.b.) and represent the mean ± the SD of at least three independent experiments.

### 2.8. Volatile Compound Analysis

The volatile organic compounds (VOCs) were analyzed using the headspace solid-phase micro-extraction technique (HS-SPME) and gas chromatography–mass spectrometry (GC–MS) according to the methodology suggested by Sánchez-Adriá et al. [41]. Volatile compounds were identified and confirmed by comparing the obtained mass spectral data with those in the NIST (National Institute of Standards and Technology) library, using a match quality threshold greater than 80%. Peak areas were normalized to the peak area of the internal standard, and the relative abundance of each volatile compound was calculated as a percentage of the total peak area in the chromatogram. The values represent the mean ± the SD of at least three independent experiments.

### 2.9. Total Phenols Assay

One hundred milligrams of fresh sourdough was mixed with 1 mL of a methanol/water solution (80:20, *v/v*). The resulting slurry was vortexed for 1 min, incubated in an ultrasonic bath (Selecta Ultrasons, J.P. Selecta, Barcelona, Spain) for 20 min, and then placed in a Thermomixer 5436 (Eppendorf, Hamburg, Germany), where it was agitated in the dark at 500 rpm and 25 °C for 1 h. Finally, the mixture was centrifuged at 12,000× *g* at 4 °C for 15 min, and the supernatant was collected and processed immediately for phenol and antioxidant activity determination.

The phenolic content was analyzed using the Folin–Ciocalteu method [42]. Briefly, sourdough extracts were diluted in water (40- to 80-fold dilution), and 250 µL of the diluted extract was mixed with 100 µL of a 10-fold aqueous dilution of Folin–Ciocalteu reagent (cat# F9252, Sigma-Aldrich, Darmstadt, Germany). The mixture was equilibrated at room temperature for 10 min, followed by the addition of 0.3 mL of Na_2_CO_3_ (10%, *w/v*). The reaction was incubated in the dark at 40 °C for 30 min. The absorbance was measured at 725 nm using a UV–vis spectrophotometer (UV-1900 model, Shimadzu Corp., Kyoto, Japan), and the values were compared against a standard curve of gallic acid (up to 25 µg/mL) prepared from an ethanol-based stock solution (80 mg/mL). The results are expressed as milligrams of gallic acid equivalents (GAE) per gram of sourdough (f.b.) and represent the mean ± the SD of at least three independent experiments.

### 2.10. Antioxidant Activity

The antioxidant activity assay of the sourdough extracts was performed using the DPPH (2,2-diphenyl-1-picrylhydrazyl; cat# 1898-66-4, Sigma-Aldrich, St. Louis, MO, USA) method [43], as described by Datta et al. [44] with slight modifications. Briefly, a daily working solution of 100 µM DPPH was prepared from an 8 mM DPPH stock solution (in methanol), and 500 µL of the working solution was mixed with 250 µL of diluted sourdough extract or a Trolox (cat# SC-200810, Santa Cruz Biotechnology, Santa Cruz, CA, USA) solution. In all cases, 80% methanol was used as the dilution solvent. The mixture was incubated in the dark at room temperature for 30 min, and its absorbance at 517 nm was recorded against that of the solvent. A blank was prepared by substituting the test sample or standard with 80% methanol, and its absorbance was used to calculate the scavenging activity, expressed as the inhibition ratio (%) = [(the blank absorbance − the sample or standard absorbance)/the blank absorbance] × 100.

For the EC50 calculation for the sourdough extracts, the procedure described by Xiao et al. [45] was followed. Inhibition ratios (Y) were plotted against concentrations of diluted sourdough extracts (X). Two concentrations enclosing a 50% inhibition ratio were selected, and the EC50 (µg/mL) was calculated by substituting the value of Y with 50 in the regression equation Y = AX + B. For the calculation of the Trolox Equivalent Antioxidant Capacity (TEAC) of the samples, calibration curves with an R^2^ > 0.99 were generated using six Trolox concentrations in the range of 0.01–0.06 mM prepared from a 50 mM stock solution (in ethanol). The antioxidant capacity was expressed as the μmols of Trolox equivalents (TEAC) per gram of sourdough (f.b.). The values represent the mean ± the SD of at least three independent experiments.

### 2.11. Leavening Ability

An automated multi-channel AF-1101-10W Fermograph II gas monitor (ATTO Corporation, Tokyo, Japan) was used to evaluate the leavening ability of sourdough. For this, bread dough samples were prepared by mixing 100 g of flour, 20 g of sourdough, and 60 mL of 1.6% NaCl. The resulting dough was divided into pieces (50 g), placed in a pre-warmed bottle, and incubated in a 30 °C water bath while being gently shaken (80 rpm). The amount of CO_2_ evolved was recorded at 30 min intervals for 18 h from the fermentation onset. The values are expressed as the mL of CO_2_ per g of bread dough (f.b.) and represent the mean ± the SD of at least three independent experiments.

### 2.12. Statistical Analysis

The statistical methodology used in this study was carried out using R software [46,47]. The analysis involved two main steps: comparisons between two formulations and comparisons between treatments within each formulation.

First, two-sample *t*-tests were performed to compare the two formulations (C and CW) for each sample, using a 95% confidence level. A repeated measures ANOVA was then used to assess the differences between treatments within each formulation, accounting for the repeated measures design by including replicates as a subject identifier and treatments as a within-subject factor. For significant ANOVA results, post hoc pairwise comparisons were performed using paired *t*-tests.

## 3. Results and Discussion

### 3.1. Sourdough Formulation, Flours and Starter Selection

A sourdough prepared with roasted and milled carob flour, the most common form of carob pod consumption, was analyzed. Alternatively, a blend of flours, created by replacing 50% of the carob flour with wheat flour, was used to improve the nutritional composition of the sourdough. As reported, carob flour contains low amounts of easily assimilable nitrogen [48], which is essential for optimal microbial activity, particularly for lactic acid bacteria (LAB) [49]. To address this limitation, a high-strength wheat flour (33% gluten), suitable for long fermentation processes, was selected. The flour was mixed with tap water, resulting in a dough yield [DY = (flour mass + water mass) × 100/flour mass] of 250, a value representative of soft sourdoughs (DY > 200). A higher dough yield promotes the activity of enzymes and microorganisms, accelerating acidification compared to firm doughs (DY < 200) [50]. As expected, the water activity (a_w_) values of carob and carob–wheat sourdough were lower, 0.971 ± 0.000 and 0.983 ± 0.001, respectively, than those found for a control sourdough (DY of 250) prepared with 100% wheat flour, 0.994 ± 0.001. Nevertheless, the a_w_ of carob sourdough remained within the range that supports the growth of most LAB and yeast species [51].

Regarding the microbial counts, the flours exhibited low cell densities, below 3.0 log_10_ cfu/g (f.b.), for both yeast and LAB and thus made a minimal contribution to the microbial load of the sourdoughs prior to inoculation. For this purpose, a pool of mixed microorganisms composed of three yeast species (*S. cerevisiae*, *K. humilis*, and *T. delbrueckii*) and three LAB species (*L. plantarum*, *F. sanfranciscensis*, and *L. helveticus*), isolated from artisanal sourdough bakeries in our laboratory [33] and common to worldwide sourdoughs [21,52,53], was used as the initial starter. This approach aimed to generate microbial diversity and to determine the ability of each species to outcompete other yeast and LAB species in carob and carob–wheat matrices. A mature sourdough often harbors only one or two dominant microbial species [33,34,35,36,37,38,39,40,41] as a result of differences in their inherent ability to adapt to this specific environment and the existence of positive and negative interactions among species [54,55].

### 3.2. Sourdough Fermentation, pH and TTA

A type III sourdough was used, in which the fermentation process is initiated by adding a starter culture to the flour–water mixture, followed by traditional backslopping [46]. Six successive refreshments (R1–R6) were carried out, followed by a fermentation period at 25 °C for 24 h (_24), except for in the final dough (R6), where fermentation was extended to 48 h (_48). Samples of unfermented control carob (C0) and carob–wheat (CW0) sourdoughs, along with fermented R3_24, R6_24, and R6_48 sourdoughs, were analyzed to determine their key technological and biochemical parameters.

As shown in Table 1, the initial pH of the control carob sourdough (4.79 ± 0.05) remained relatively stable throughout the backslopping process (CR6_48; pH 4.65 ± 0.13). In contrast, the pH of the carob–wheat flour–water mixture decreased significantly after just three refreshments, dropping from an initial pH of 4.95 ± 0.06 to 4.40 ± 0.08 in CWR3_24 (*p* < 0.05). Then, no significant changes were observed, except in CWR6_48, where an additional pH decrease to around 4.0 (*p* < 0.05) was noted (Table 1). Consistent with this, the TTA values in carob sourdoughs showed no significant variations during backslopping, whereas in carob–wheat dough, they increased gradually from an initial value of 3.94 ± 0.09 to 8.1 ± 0.5 mL of 0.1 N NaOH (*p* < 0.05) for the CWR6_48 sample.

### 3.3. By-Product Profile

Sourdough samples were further analyzed to assess the accumulation of fermentation by-products during the backslopping process. Heterofermentative LAB species, such as *L. plantarum* and *F. sanfranciscensis*, produce a mixture of lactic acid, acetic acid, and/or ethanol. In contrast, ethanol and glycerol are the main by-products of yeast fermentative activity [56].

As shown in Table 1, the levels of lactic and acetic acids in carob sourdough samples were below the detection limit, consistent with the absence of significant pH and total titratable acidity (TTA) variations throughout the backslopping process. Similarly, acetic acid was undetectable in carob–wheat sourdoughs. However, lactic acid accumulation in these samples became evident after the third refreshment, reaching 8.6 ± 0.9 mg/g sourdough (f.b.) in CWR6_48 (Table 1), a value close to the reported mean lactic acid concentration in bakery sourdough [53]. This suggests that the incorporation of wheat flour, a source of assimilable nitrogen and essential nutrients, into the carob sourdough formulation stimulated LAB fermentative activity. Additionally, the bacterial composition of our starter culture, particularly the absence of acetic acid bacteria [55], along with the fermentation conditions employed—such as a high hydration level and warm temperature (25 °C), which favor lactic acid production over acetic acid production [57]—may have influenced the levels of organic acids detected.

Unlike for lactic acid, the ethanol concentrations at the end of the backslopping process were similar in carob- and carob–wheat-based doughs, but ethanol accumulation was faster in carob–wheat samples (Table 2). This result could be attributed to the higher metabolic activity of yeasts. For instance, the ethanol levels were 24.0 ± 2.2 mg/g and 13.6 ± 0.08 mg/g sourdough (f.b.) in CWR3_24 and CR3_24, respectively. In contrast, glycerol accumulation followed a different trend, with higher values in 100% carob sourdough (Table 2). This is consistent with the lower a_w_ of this sourdough, as glycerol—produced at the expense of ethanol—plays a key role as an osmoprotectant in yeasts [58].

### 3.4. Soluble Sugars

We analyzed the soluble sugar variations during sourdough fermentation. As expected, C0 had about twice the sugar content of CW0, with sucrose as the predominant sugar, while maltose appeared only in wheat-containing sourdough (Table 2). We also detected D-pinitol (3-O-methyl-D-chiro-inositol), a known antioxidant in carob beans [59], at 25.5 ± 1.9 mg/g (f.b.) in C0 and about half that (12.6 ± 0.9 mg/g) in CW0 (Table 2). These results align with the known composition of carob and wheat flours [60,61] and their proportions in each sourdough formula.

As expected, the soluble sugar levels in sourdough decreased with repeated fermentation. The reduction was faster in wheat-containing sourdough (Table 2), corresponding to higher LAB counts (Table 1). The dynamics of individual sugars also varied in carob and carob–wheat sourdoughs, likely reflecting the evolution of the sourdough microbiota (Table 1), its enzymatic activities regarding carbohydrates, and its preference for different carbon sources.

The sucrose levels depended, among other factors, on the activity of yeast invertase, which cleaved the disaccharide into glucose and fructose [56], whereas the amount of maltose consistently remained low, as it was rapidly depleted by LAB during fermentation [62]. Differences in metabolic capabilities related to carbohydrate utilization between microbial species—often strain-dependent—may also explain the variations in the sugar levels. For example, *F. sanfranciscensis* strains can be classified into different groups based on their ability to utilize glucose and fructose as sole carbon sources [62]. *S. cerevisiae* is known for its flexibility in carbon source utilization [56], including the usage of both di- and monosaccharides, whereas *K. humilis* cannot ferment maltose [63].

Despite these differences, the entire backslopping process in carob–wheat sourdough led to a 90–98% reduction in the total soluble sugar content (Table 2), with nearly a 60% reduction in carob sourdough (Table 2). Furthermore, there was no significant variation in the pinitol content in either carob or carob–wheat sourdough (Table 2), a result that aligns well with previous studies on the submerged fermentation of carob pods by yeasts [64].

### 3.5. Counting and Sequencing

We examined the dynamics of LAB and yeast species in carob and carob–wheat sourdoughs using culture-dependent and -independent methods. First, we evaluated the number of cultivable LAB and yeasts present in the sourdough samples (Table 1). Both the C0 and CW0 samples showed comparable cell counts, with values of around 5.0 log_10_ cfu/g sourdough (f.b.) for both LAB and yeast. During the first three refreshments, the number of yeasts in both sourdough types increased to >7 log_10_ cfu/g and then hardly varied with additional refreshments or fermentation time (Table 1). Regardless of the LAB population, the cell counts were around an order of magnitude higher in carob–wheat than carob sourdough after the third refreshment, 8.58 ± 0.04 versus 6.99 ± 0.18 log_10_ cfu/g, respectively, a difference that was maintained throughout the rest of the backslopping process (Table 1). This result confirms the increased growth and metabolic activity of LAB caused by the addition of wheat flour to the sourdough formula.

**Figure 1:**
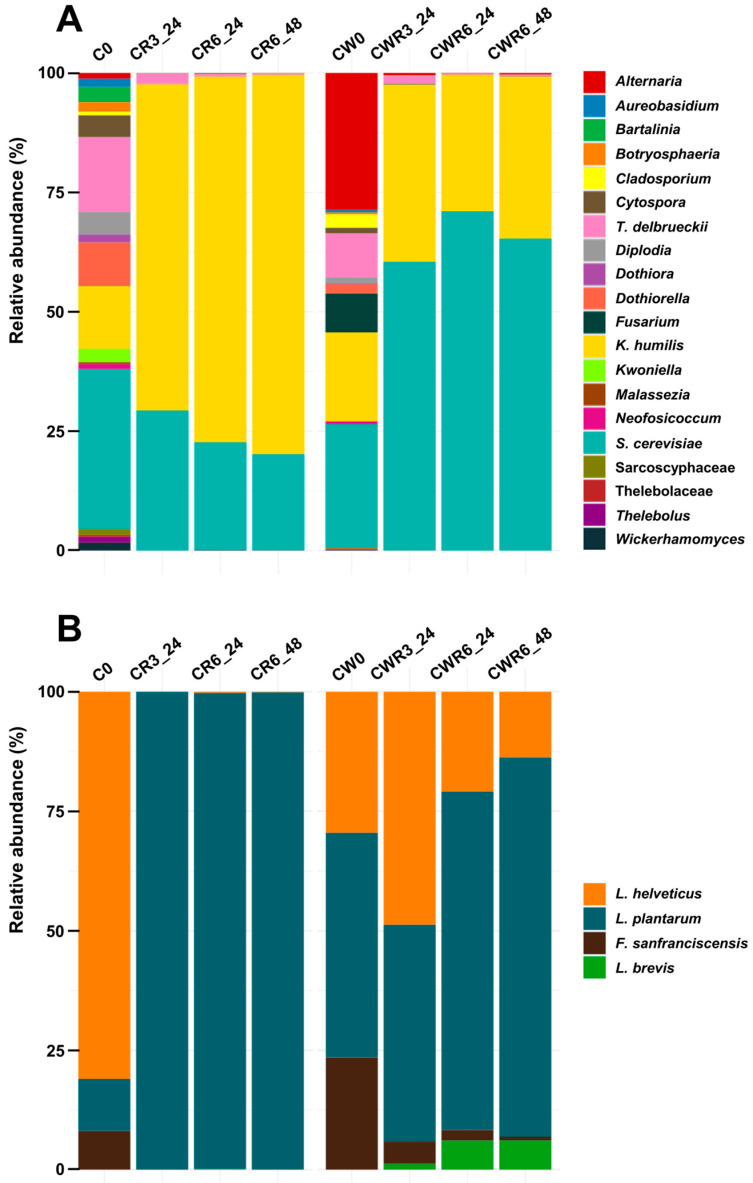
Relative abundance of microbial communities during sourdough fermentation. Stacked column plots depict changes in the relative abundance of fungi (**A**) and bacteria (**B**) over the course of sourdough fermentation. Fungal communities, including the yeasts *S. cerevisiae*, *K. humilis*, and *T. delbrueckii*, and lactic acid bacteria (LAB), specifically *L. plantarum*, *F. sanfranciscensis*, and *L. helveticus*, were used as a starter pool. Control sourdoughs made from carob flour (C0) and carob–wheat blends (CW0) were fermented for 24 h at 30 °C (denoted as _24), followed by six serial backslopping steps under the same conditions. In the final refreshment step, fermentation was extended to 48 h (_48). The dynamics of microbial composition changes were assessed after the 3rd (R3) and 6th (R6) refreshments. For further details, refer to the Section 2.

Next, we analyzed the microbial changes throughout backslopping using a procedure based on Next-Generation Sequencing (NGS). As can be seen, the fungal community in our initial sourdough samples, C0 and CW0, was diverse, with at least 20 amplicon sequence variants (Figure 1A). As anticipated, the inoculated species *S. cerevisiae*, *K. humilis*, and *T. delbrueckii* were dominant, accounting for approximately 50–70% of the total reads. The remaining species, mostly present at a low abundance—with the exception of species from the genus *Alternaria*, which is notably prevalent in wheat flour [65]—were common environmental molds, plant pathogens, and plant endophytes [55]. Nevertheless, all of them vanished after the third refreshment, and it was almost only *K. humilis* and *S. cerevisiae* that persisted in CR3_24 and CWR3_24 and throughout the full backslopping process. Notably, *K. humilis* was enriched in carob sourdough, while *S. cerevisiae* was prevalent in carob–wheat samples (Figure 1A). This phenomenon likely occurred because *S. cerevisiae* preferentially utilizes maltose, while *K. humilis* displays a maltose-nonfermenting phenotype. Indeed, studies of yeast–LAB interactions in sourdough have suggested that maltose is the driver of *S. cerevisiae* growth [66]. On its part, the decline in the reads of *T. delbrueckii* was remarkable and suggests that the growth of *S. cerevisiae* and *K. humilis* altered its ability to proliferate in carob-based sourdough. Consistent with this, a negative co-persistence pattern between *S. cerevisiae* and *T. delbrueckii* has been proposed [67,68].

Concerning LAB, the sequence reads in both sourdoughs were assigned mainly to the three bacterial species used in our inoculum. Subsequently, the evolution of the LAB community was characterized by the dominance of *L. plantarum*, particularly in carob sourdough, where it represented almost 100% of the reads after three fermentation cycles (Figure 1B). In carob–wheat sourdough, this species was prevalent, together with *L. helveticus*, but the predominance of the former increased in subsequent refreshments, reaching 85% of the relative abundance in CWR6_48.

To our surprise, *F. sanfranciscensis* could not persist in carob sourdough, and its relative abundance in carob–wheat sourdough was progressively reduced over the fermentation cycles. *F. sanfranciscensis* is the dominant bacterium in most sourdoughs, quite often co-occurring with *K. humilis* [55]. The antimicrobial activity of polyphenol-enriched carob flour [69] could account, at least in part, for the inability of this bacterium to proliferate in our sample set. Evidence of negative interactions between *F. sanfranciscensis* and *S. cerevisiae* or *L. plantarum* has also been reported [55].

Finally, we observed the progressive establishment of *Levilactobacillus brevis* in carob–wheat sourdough (Figure 1B). This LAB and *L. plantarum* were reported to be the most commonly observed pair of co-occurring bacteria in a global study on sourdough biodiversity [51]. In conclusion, the maltose availability and polyphenol levels, along with both negative and positive interactions between LAB and yeast species, appear to have influenced the evolution of the sourdough microbiota over the six fermentation cycles.

### 3.6. Leavening Ability of Sourdough

The leavening capacity of carob and carob–wheat sourdoughs was assessed after six refreshment cycles. Gas production by yeasts and heterofermentative lactic acid bacteria (LAB) during fermentation is critical for achieving the optimal bread volume and desirable organoleptic properties [70]. To evaluate this, bread dough was prepared using 20% sourdough (g per 100 g flour) without the addition of commercial yeast, and the CO_2_ production was monitored over an 18 h period.

**Figure 2:**
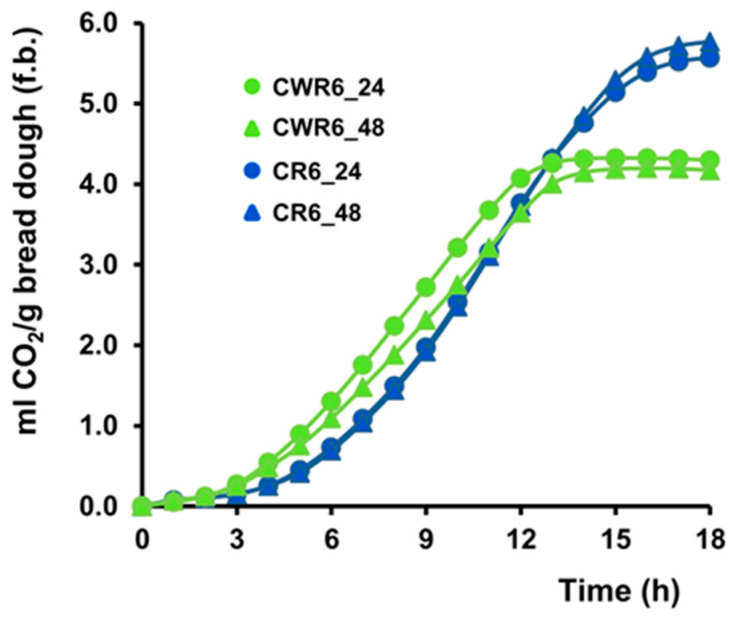
Leavening activity of sourdough. Bread dough samples containing 20% carob (CR6_24 and CR6_48) or carob–wheat (CWR6_24 and CWR6_48) sourdough were analyzed to determine their leavening ability by using a Fermograph II gas monitor. The amount of CO_2_ produced was recorded at 60 min intervals for 18 h from the fermentation onset. Values are expressed as the mL of CO_2_ per g of bread dough (f.b.) and represent the mean ± the SD of at least three independent experiments.

As shown in Figure 2, both sourdoughs exhibited good leavening activity. However, the carob–wheat sourdough produced CO_2_ more rapidly at 30 °C, likely due to yeasts’ adaptation to maltose, the main sugar present in wheat flour. After 6 h, the dough containing CWR6_24 generated approximately 1.30 mL CO_2_/g (fresh basis), compared to 0.75 mL/g for CR6_24. The gas production then gradually declined between 12 and 15 h, reaching approximately 4.0 mL/g for the carob–wheat dough and 5.5 mL/g for the carob dough after 18 h, possibly due to the higher sucrose content in the latter. Finally, the sourdough fermentation time (24 vs. 48 h) had no significant effect on the fermentative performance of the bread dough (Figure 2), consistent with the similar yeast cell counts observed in these samples (Table 1).

### 3.7. Antioxidant Activity and Polyphenol Content

**Figure 3:**
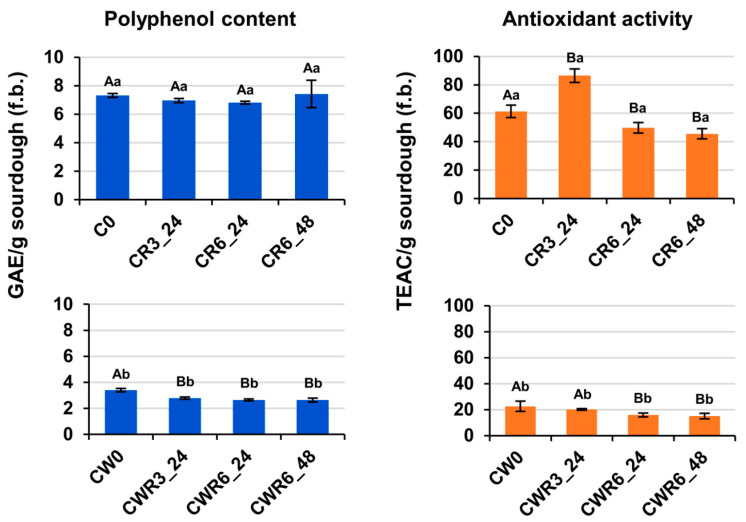
Dynamics of antioxidant activity and polyphenol content during sourdough fermentation. Methanol/water extracts from sourdough samples described in Figure 1 were analyzed to determine their polyphenol content and antioxidant activity using Folin–Ciocalteu [42] and DPPH [43] methods, respectively. Results are expressed as milligrams of gallic acid equivalents (GAE) per gram of sourdough (f.b.) for polyphenols and as micromoles of Trolox equivalents (TEAC) per gram of sourdough (f.b.) for antioxidant activity. Data represent mean values (±SD) from three independent replicates. Different superscript capital letters indicate significant differences (*p* < 0.05) between backslopped samples and their corresponding control within each sourdough type (C or CW). Different superscript lowercase letters denote significant differences (*p* < 0.05) between sourdough types at the same backslopping step.

We analyzed the polyphenol content and antioxidant activity in methanol/water extracts from sourdough samples. As shown in Figure 3, carob flour sourdough (C0) had the highest extractable polyphenol content (7.67 ± 0.71 mg GAE/g, f.b.), while carob–wheat sourdough (CW0), with half the amount of carob flour, contained 3.40 ± 0.12 mg GAE/g.

The polyphenol levels in wheat flour typically range from 0.29 to 0.50 mg GAE/g [71] and are even lower in refined flour [72]. In contrast, carob flour is rich in phenolic compounds, mainly gallic acid [73], making it the key source of these compounds in carob-based bread [30,31]. Accordingly, the DPPH scavenging activity, expressed as the TEAC, was about twice as high in carob sourdough as in carob–wheat sourdough (Figure 3), as the presence of phenolic compounds largely determines carob flour’s antioxidant capacity [30].

Fermentation slightly but significantly (*p* < 0.05) reduced the polyphenol content and antioxidant activity in carob–wheat sourdough. After six backslopping cycles and 48 h at 25 °C (CWR6_48), these values dropped by 22% and 33%, respectively, compared to those of CW0 (Figure 3). In carob sourdough, the antioxidant activity initially increased by ~40% in CR3_24 but ultimately declined by 25% by the process’s end, while the extractable polyphenol levels remained unchanged (Figure 3).

These results contrast with studies reporting increased antioxidant activity in fermented plant-based foods [74]. LAB-driven fermentation has been shown to enhance the polyphenol content in wheat [75] and legume flours [28], including carob flour [30]. However, those studies used monocultures of LAB in single-step fermentations (~24 h), whereas our process involved repeated cycles and competition between yeasts for sugars, likely affecting the polyphenol evolution and antioxidant activity.

### 3.8. Volatile Profile

**Figure 4:**
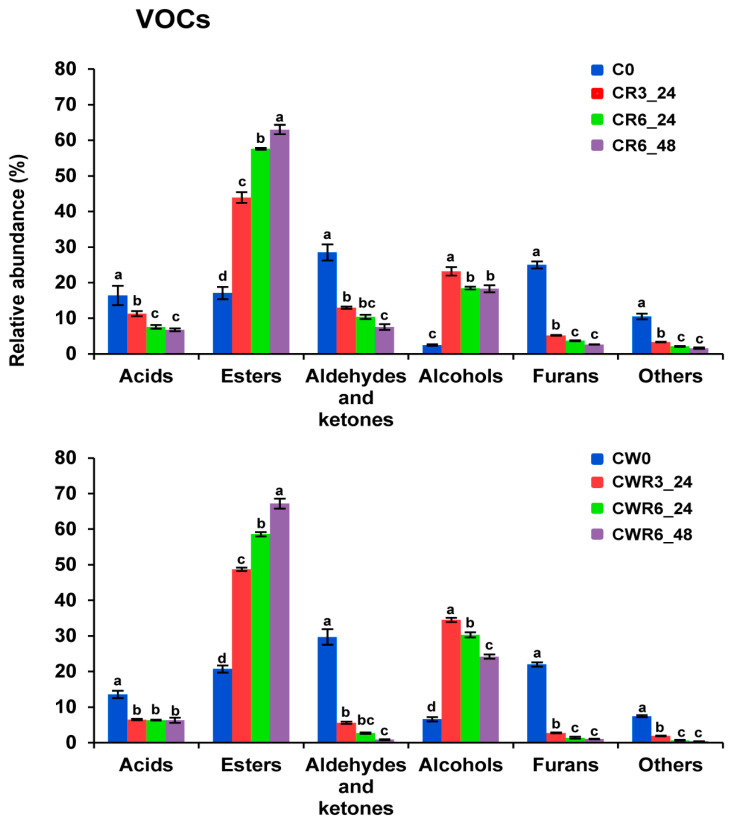
Abundance of volatile families in sourdough. The relative abundance of VOC families in the carob (upper graph) and carob–wheat (lower graph) sourdough samples described in Figure 1 was calculated as the sum of the percentages of the corresponding individual species (see Tables S1 and S2). Different superscript lowercase letters indicate significant differences (*p* < 0.05) between the backslopped samples and their corresponding control within each sourdough type (C or CW). The data represent the mean values (±the SD) from three independent replicates.

Seventy volatile organic compounds (VOCs) were extracted and identified using SPME-GC-MS for carob (Table S1) and carob–wheat (Table S2) sourdough samples. As illustrated in Figure 4, no major differences were observed in the relative abundance of VOC families between the control carob and carob–wheat sourdough samples. Esters represented the most diverse chemical class, comprising 24 to 28 molecular species. However, aldehydes, ketones, and furans were the most abundant compounds, collectively accounting for more than 50% of the relative content in both C0 and CW0 samples (Figure 4).

The high content of these volatile families aligns with the use of toasted carob flour in sourdough, serving as the primary source of VOCs, with 2-methyl-butanal and furfural being the predominant compounds (Tables S1 and S2). Toasting is a common practice aimed at reducing residual moisture and the microbial load while enhancing the sensory attributes of carob flour compared to those of raw powder [76]. This process promotes the formation of Maillard reaction products, such as furfural [77]. Furthermore, toasting decreases the abundance of acids, esters, and alcohols, which are typically the most abundant in raw carob flour [78]. For example, 2-methyl-propanoic acid, known for its characteristic cheesy, acidic, and buttery aroma in carob pods and comprising 47–48% of the relative VOC content [79], represented only 8–9% of the VOCs in C0 (Table S1) and CW0 (Table S2) samples.

Significant variations in the relative abundance of volatile families were observed during sourdough fermentation. As shown in Figure 4, the abundance of esters and alcohols increased notably with the progressive backslopping of both carob and carob–wheat sourdoughs, with 2- and 3-methyl-1-butanol, phenylethyl alcohol, ethyl 2-methylpropionate, methyl 2-methylbenzoate, and, in particular, ethyl hexanoate, the abundance of which ranged between 36 and 39% of the total VOCs, being the major molecular species (Tables S1 and S2). The identified alcohols and esters, likely formed via the Erhlich pathway [80] and the acetyltransferases-catalyzed reaction [81] in the yeast cells, are the most abundant volatile compounds reported in direct baking dough [82,83].

Unlike for esters and alcohols, sourdough fermentation reduced the relative abundance of acids, aldehydes, ketones, and furans (Figure 4). The reduction in aldehydes and furans was especially dramatic, with major species in the control sourdough such as 2-methyl butanal or furfural, representing around 8–10 and 13% of the total VOCs, respectively, being non-detectable after just three cycles of backslopping (Tables S1 and S2). A decline in acids and aldehydes has been reported in yeasted baking dough [81]. In addition, volatile compounds, such as hexanal, acetaldehyde, hexanoic acid, 1-hexanol, 1-pentanol, 2-pentylfuran, and dimethylsulfide, derived mainly from LAB activity [84], were not detected or their relative content decreased during the fermentation process (Tables S1 and S2). Altogether, these results emphasize the importance of yeast metabolism in the generation of VOCs in our samples and the minor role of LAB, as has been previously reported for wheat sourdough [81].

## 4. Conclusions

This study demonstrates that prolonged sourdough fermentation with multiple refreshments significantly enhances the functional properties of carob flour for baking applications. The incorporation of wheat flour promotes microbial activity—particularly that of lactic acid bacteria (LAB)—resulting in effective sugar reduction, acidification, and the development of a distinctive sourdough microbiota. In particular, *K. humilis* and *S. cerevisiae*, two yeast species commonly found in sourdough, along with *L. plantarum*, were identified as the dominant species in carob- and carob–wheat-based sourdoughs. Beyond their relevance for understanding microbial interactions in the sourdough ecosystem, these findings could support the development of novel microbial starter cultures for the food industry.

Dynamic microbial interactions and the modulation of volatile organic compounds (VOCs) during fermentation contributed to the improved sensory and technological characteristics of carob-based sourdough. Although a slight reduction in antioxidant activity was observed, key nutritional compounds such as pinitol were retained. These findings underscore the potential of carob-based sourdough as a sustainable and health-promoting ingredient for the food industry, addressing both nutritional and technological challenges.

Mature sourdoughs, maintained through continuous propagation in bakeries, are routinely used as leavening agents or baking improvers to produce high-quality baked goods. Accordingly, the results of this study can be readily adopted by artisanal bakers and commercial producers. Sourdough fermentation imparts a depth of flavor typically absent in high-temperature, short-duration fermentation processes. As demonstrated, prolonged fermentation not only mitigates the limitations of carob flour but also enhances its baking performance. These insights may facilitate the broader use of carob flour and support innovation in the utilization of underexploited crops.

## Figures and Tables

**Table 1:** Chemical traits and cell counts of sourdoughs *.

			Organic Acids (mg/g Sourdough) ^3^		log_10_ cfu/g Sourdough ^3^	
Sourdough ^1^	pH	TTA ^2^	Lactic Acid	Acetic Acid	LAB ^4^	Yeast
C0	4.79 ± 0.05 ^Aa^	6.4 ± 0.6 ^Aa^	n.d. ^Aa^	n.d.	5.0 ± 0.2 ^Aa^	5.19 ± 0.02 ^Aa^
CR3_24	4.69 ± 0.04 ^Aa^	6.4 ± 0.6 ^Aa^	n.d. ^Aa^	n.d.	6.99 ± 0.18 ^Ba^	7.33 ± 0.08 ^Ba^
CR6_24	4.71 ± 0.12 ^Aa^	5.8 ± 0.7 ^Aa^	n.d. ^Aa^	n.d.	7.7 ± 0.2 ^Ba^	7.41 ± 0.02 ^Ba^
CR6_48	4.65 ± 0.13 ^Aa^	6.4 ± 0.7 ^Aa^	n.d. ^Aa^	n.d.	7.9 ± 0.2 ^Ba^	7.410 ± 0.008 ^Ba^
CW0	4.95 ± 0.06 ^Ab^	3.94 ± 0.09 ^Ab^	n.d. ^Aa^	n.d.	5.2 ± 0.2 ^Aa^	5.27 ± 0.12 ^Aa^
CWR3_24	4.40 ± 0.08 ^Bb^	5.4 ± 0.3 ^Bb^	6.3 ± 1.05 ^Bb^	n.d.	8.58 ± 0.04 ^Bb^	7.60 ± 0.07 ^Ba^
CWR6_24	4.30 ± 0.07 ^Bb^	6.1 ± 0.4 ^Ba^	7.4 ± 0.7 ^Bb^	n.d.	8.69 ± 0.17 ^Bb^	7.61 ± 0.02 ^Ba^
CWR6_48	4.04 ± 0.12 ^Bb^	8.1 ± 0.5 ^Bb^	8.6 ± 0.9 ^Bb^	n.d.	8.76 ± 0.15 ^Bb^	7.54 ± 0.08 ^Ba^

* The data represent the mean value (± the SD) from three independent replicates. Different superscript capital letters indicate significant differences (*p* < 0.05) between backslopped samples and their corresponding control within each sourdough type (C or CW). Likewise, different superscript lowercase letters denote significant differences (*p* < 0.05) between sourdough types at the same backslopping step. ^1^ Control carob (C0) and carob–wheat (CW0) sourdoughs were fermented for 24 h (_24) at 30 °C, followed by six backslopping steps (R1 to R6) under the same conditions, except for in the final refreshment, where fermentation was extended to 48 h (_48). Only data from the control, R3, and R6 sourdoughs are shown. For more details, see the Section 2. ^2^ Total titratable acidity. ^3^ Fresh basis. ^4^ Lactic acid bacteria.

**Table 2:** Sugar content in sourdoughs *.

	Compounds (mg/g Sourdough) ^2^						
Sourdough ^1^	Pinitol	Glucose	Fructose	Sucrose	Maltose	Glycerol	Ethanol
C0	25.5 ± 1.9 ^Aa^	10.8 ± 1.3 ^Aa^	14.9 ± 2.3 ^Aa^	100.8 ± 7.7 ^Aa^	n.d. ^Aa^	n.d. ^Aa^	n.d. ^Aa^
CR3_24	29.2 ± 2.3 ^Aa^	2.1 ± 0.6 ^Ba^	8.08 ± 1.12 ^Aa^	94.77 ± 5.03 ^Aa^	n.d. ^Aa^	6.6 ± 0.7 ^Ba^	13.6 ± 0.8 ^Ba^
CR6_24	29.1 ± 2.9 ^Aa^	5.0 ± 1.3 ^Ba^	9.8 ± 2.3 ^Aa^	84.34 ± 1.05 ^Aa^	n.d. ^Aa^	7.2 ± 1.3 ^Ba^	15.2 ± 0.7 ^Ba^
CR6_48	29.9 ± 0.9 ^Aa^	5.7 ± 1.2 ^Ba^	8.06 ± 0.6 ^Ba^	41.3 ± 7.3 ^Ba^	n.d. ^Aa^	9.7 ± 0.9 ^Ba^	26.3 ± 3.4 ^Ba^
CW0	12.6 ± 0.9 ^Ab^	5.0 ± 0.9 ^Ab^	7.3 ± 0.8 ^Ab^	45.3 ± 3.6 ^Ab^	1.0 ± 0.4 ^Aa^	n.d. ^Aa^	n.d. ^Aa^
CWR3_24	13.8 ± 0.8 ^Ab^	2.0 ± 0.3 ^Ba^	4.6 ± 0.4 ^Bb^	16.0 ± 1.3 ^Bb^	0.16 ± 0.06 ^Bb^	5.1 ± 0.6 ^Bb^	24.0 ± 2.2 ^Bb^
CWR6_24	14.3 ± 0.5 ^Ab^	1.86 ± 0.07 ^Bb^	5.0 ± 0.9 ^Bb^	10.1 ± 2.5 ^Bb^	0.12 ± 0.03 ^Bb^	5.2 ± 0.6 ^Ba^	24.9 ± 1.3 ^Bb^
CWR6_48	14.6 ± 0.4 ^Ab^	0.55 ± 0.09 ^Bb^	0.5 ± 0.2 ^Bb^	0.63 ± 0.16 ^Bb^	n.d. ^Ba^	5.6 ± 0.2 ^Bb^	31.8 ± 1.4 ^Ba^

* The data represent the mean value (± the SD) from three independent replicates. Different superscript capital letters indicate significant differences (*p* < 0.05) between backslopped samples and their corresponding control within each sourdough type (C or CW). Likewise, different superscript lowercase letters denote significant differences (*p* < 0.05) between sourdough types at the same backslopping step. ^1^ Control carob (C0) and carob–wheat (CW0) sourdoughs were fermented for 24 h (_24) at 30 °C, followed by six backslopping steps (R1 to R6) under the same conditions, except for in the final refreshment, where fermentation was extended to 48 h (_48). Only data from the control, R3, and R6 sourdoughs are shown. For more details, see the Section 2. ^2^ Fresh basis.

## Data Availability

Raw sequencing data are available in the European Nucleotide Archive (ENA) under the accession number PRJEB88591. The rest of the original contributions presented in this study are included in the article; further inquiries can be directed to the corresponding author.

## Supplementary Materials

The following supporting information can be downloaded at https://www.mdpi.com/article/10.3390/foods14101677/s1: Table S1: Main volatile organic compounds (VOCs) in carob sourdough; Table S2: Main volatile organic compounds (VOCs) in carob–wheat sourdough.
