# Supplementary material for: The Effects of Sourdough Fermentation on the Biochemical Properties, Aroma Profile and Leavening Capacity of Carob Flour
Source: Foods. 2025 May 9;14(10):1677. doi: 10.3390/foods14101677 (PMC12111545; doi:10.3390/foods14101677)
Supplement: Supplementary file 1 [file foods-14-01677-s001.zip › Table S1.pdf]

Table S1. Main volatile organic compounds (VOCs) in carob sourdough*

COMPOUND	Sourdough[1]			
	C0	CR3_24	CR6_24	CR6_48
**ACIDS**				
Acetic acid	0.8±0.3[a]	n.d.	n.d.	n.d.
Propanoic acid, 2-methyl-	8.2±2.1[a]	5.7±0.2[ab]	5.5±0.6[ab]	3.55±0.06[b]
Butanoic acid	0.63±0.04[a]	0.46±0.09[b]	0.47±0.05[b]	0.38±0.03[b]
Butanoic acid, 2-methyl-	0.46±0.15[a]	n.d.	n.d.	n.d.
Hexanoic acid	5.8±0.7[a]	4.4±0.3[b]	1.61±0.10[c]	2.1±0.3[c]
Octanoic acid	0.53±0.10[b]	0.85±0.15[a]	n.d.	0.74±0.09[ab]
**TOTAL Acids**	**16.4±2.7[a]**	**11.3±0.7[b]**	**7.6±0.5[c]**	**6.8±0.4[c]**
**ESTERS**				
Ethyl acetate	n.d.	n.d.	0.50±0.04[a]	0.54±0.10[a]
Methyl isobutyrate	0.90±0.14[a]	n.d.	n.d.	n.d.
Propanoic acid, 2-methyl-, ethyl ester	n.d.	3.8±0.2[c]	6.10±0.02[a]	5.3±0.3[b]
Butanoic acid, ethyl ester	n.d.	1.51±0.08[b]	2.91±0.06[a]	2.79±0.10[a]
Butanedioic acid, monomethyl ester	0.41±0.06[a]	n.d.	n.d.	n.d.
Isobutiric acid, butyl ester	1.5±0.4[bc]	2.11±0.10[a]	1.89±0.02[ab]	1.37±0.09[c]
Hexanoic acid, methyl ester	3.4±0.5[a]	2.67±0.15[a]	0.94±0.15[b]	0.97±0.13[b]
Hexanoic acid, ethyl ester	n.d.	26.1±1.0[c]	35.1±0.2[b]	39.2±0.6[a]
Propanoic acid, 2-methyl-, 3-methylbutyl ester	0.88±0.10[c]	1.75±0.06[a]	1.35±0.02[b]	1.35±0.07[b]
Propanoic acid, 2-methyl-, 2-methylbutyl ester	0.30±0.05[d]	1.94±0.06[a]	1.45±0.02[b]	1.16±0.09[c]
Cyclohexanol, 1-methyl-4-(1-methylethyl) acetate	0.25±0.03[a]	n.d.	n.d.	n.d.
Heptanoic acid, ethyl ester	n.d.	n.d.	1.03±0.05[b]	1.12±0.04[a]
Isobutiric acid, hexyl ester	0.30±0.08[a]	n.d.	n.d.	n.d.
Hexanoic acid, 2-methylpropyl ester	n.d.	0.46±0.05[b]	0.50±0.05[ab]	0.56±0.03[a]
Butanoic acid, 2-methylhexyl ester	0.21±0.03[a]	n.d.	n.d.	n.d.
Benzoic acid, 2-butoxy, methyl ester	n.d.	2.1±0.2[c]	4.5±0.2[b]	6.9±0.2[a]
Octanoic acid, ethyl ester	0.97±0.14[a]	0.58±0.04[b]	0.46±0.04[b]	0.42±0.03[b]
2(1H)-Naphtalenone, 3,4,4a,5,6,7,8,8a-octahydro-5a-hydroxy-4aa,7,7-trimethyl acetate	0.42±0.07[a]	0.25±0.04[ab]	0.170±0.006[b]	0.21±0.14[ab]
7-Benzofuranamine, 2,3-dihydro-2,2-dimethyl	0.34±0.06[a]	n.d.	n.d.	n.d.
Nonanoic acid, ethyl ester	n.d.	n.d.	0.54±0.06[b]	0.64±0.04[a]
Isobutiric acid, benzyl ester	0.40±0.07[a]	0.30±0.03[b]	n.d.	n.d.
Butanoic acid, 1-methyloctyl ester	0.40±0.13[a]	0.152±0.013[b]	n.d.	0.12±0.02[b]
Ethanol, 2-(2-butoxyethoxy)-, acetate	0.26±0.03[a]	n.d.	n.d.	n.d.
1,1,3-Pentanediol, 2,2,4-trimethyl-, 1-isobutirate	0.35±0.07[a]	n.d.	n.d.	n.d.
Decanoic acid, ethyl ester	0.10±0.04[c]	0.145±0.014[c]	0.24±0.02[b]	0.37±0.03[a]
2,2,4-Trimethyl-1,3-pentanediol diisobutyrate	4.5±1.2[a]	n.d.	n.d.	n.d.
Cyclopentaneacetic acid, 3-oxo-2-pentyl, methylester	0.75±0.04[a]	n.d.	n.d.	n.d.
Isopropyl myristate	0.41±0.06[a]	n.d.	n.d.	n.d.
**TOTAL Esters**	**17.1±1.7[d]**	**43.9±1.5[c]**	**57.6±0.3[b]**	**63.0±1.3[a]**
**ALDEHYDES and KETONES**				
Butanal, 3-methyl-	3.4±0.9[a]	n.d.	n.d.	n.d.
Butanal, 2-methyl-	8.5±1.7[a]	n.d.	n.d.	n.d.
Acetoin	n.d.	n.d.	1.03±0.09[a]	0.81±0.08[b]
Benzaldehyde	0.54±0.04[a]	n.d.	n.d.	n.d.
Benzeneacetaldehyde	0.52±0.05[a]	n.d.	n.d.	n.d.

Table S1 (CONTINUATION)*

COMPOUND	Sourdough[1] C0	CR3_24	CR6_24	CR6_48
1H-pyrrole-2-carboxialdehyde, 1-ethyl-	0.27±0.03[a]	n.d.	n.d.	n.d.
Ethanone, 1-(1H-pyrrol-2-yl)-	3.92±0.56[a]	4.0±0.13[a]	3.2±0.3[a]	1.7±0.4[b]
4-Nonanone	n.d.	2.52±0.03[a]	2.06±0.13[b]	1.73±0.14[c]
2H-pyran-2-one, tetrahydro, -6-methyl-	n.d.	n.d.	3.09±0.08[a]	2.3±0.3[b]
2-Nonanone	6.4±0.91[a]	5.14±0.03[b]	n.d.	n.d.
Nonanal	2.1±0.4[a]	n.d.	n.d.	n.d.
3-Nonen-2-one	0.3±0.02[a]	n.d.	n.d.	n.d.
2-Dodecenal	0.28±0.02[a]	n.d.	n.d.	n.d.
2-Undecanone	1.0±0.2[a]	0.58±0.07[b]	0.44±0.02[b]	0.48±0.10[b]
2-Tridecanone	0.6±0.2[a]	0.39±0.05[a]	0.36±0.04[a]	0.30±0.05[a]
2-Pentadecanone	0.7±0.3[a]	0.37±0.02[ab]	0.26±0.06[b]	0.40±0.12[ab]
**TOTAL Aldehydes and Ketones**	**28.5±2.3[a]**	**13.0±0.3[b]**	**10.4±0.6[bc]**	**7.6±0.8[c]**
**ALCOHOLS**				
1-Propanol, 2-methyl-	n.d.	1.734±0.014[a]	1.47±0.02[b]	1.10±0.02[c]
Butanol, 3-methyl-	n.d.	8.4±0.5[a]	4.67±0.12[b]	5.2±0.2[b]
Butanol, 2-methyl-	n.d.	4.5±0.6[a]	3.7±0.9[a]	2.2±0.2[b]
2-Heptanol	0.47±0.04[b]	0.77±0.07[a]	0.78±0.05[a]	0.812±0.009[a]
1-Hexanol, 2-ethyl-	0.150±0.012[a]	n.d.	n.d.	n.d.
2-Nonanol	0.78±0.04[d]	2.91±0.04[a]	2.66±0.07[b]	2.51±0.04[c]
Phenylethyl alcohol	n.d.	4.7±0.3[b]	5.0±0.3[b]	6.3±0.6[a]
2-Undecanol	0.48±0.09[a]	0.227±0.003[b]	0.21±0.04[b]	n.d.
1-Dodecanol	0.57±0.16[a]	n.d.	n.d.	0.12±0.02[b]
**TOTAL Alcohols**	**2.5±0.2[c]**	**23.2±1.2[a]**	**18.5±0.4[b]**	**18.3±1.0[b]**
**FURANS**				
3(2H)-Furanone, dihydro 2-methyl-	0.45±0.09[a]	0.51±0.07[a]	n.d.	n.d.
Furfural	13.2±1.3[a]	n.d.	n.d.	n.d.
2-Furanmethanol	1.5±0.4[a]	1.39±0.04[ab]	1.29±0.07[ab]	0.93±0.13[b]
Ethanone, 1-(2-furanyl)-	1.11±0.10[a]	0.80±0.02[b]	0.79±0.04[b]	0.43±0.04[c]
2-Furancarboxaldehyde, 5-methyl-	1.1±0.2[a]	n.d.	n.d.	n.d.
2-Pentylfuran	0.23±0.04[a]	n.d.	n.d.	n.d.
2-Furanmethanol, acetate	0.19±0.02[a]	n.d.	n.d.	n.d.
Dihydro-3-methylene-5-methyl-2-furanone	4.3±1.5[a]	n.d.	n.d.	n.d.
Butanoic acid, 2-furanylmethyl ester	2.8±0.5[a]	2.51±0.11[a]	1.62±0.07[b]	1.292±0.108[b]
**TOTAL Furans**	**25.0±1.0[a]**	**5.21±0.10[b]**	**3.70±0.12[c]**	**2.65±0.05[c]**
**OTHERS**				
Silanediol, dimethyl-	1.2±0.3[ab]	1.46±0.04[a]	0.94±0.06[bc]	0.7±0.2[c]
Disulfide, dimethyl	3.02±0.6[a]	n.d.	n.d.	n.d.
Trisulfide, dimethyl-	3.5±0.3[a]	n.d.	n.d.	n.d.
1,3-Dioxolane, 4,4-dimethyl-2-pentadecyl-	0.22±0.03[a]	n.d.	n.d.	n.d.
1,3-Dioxolane, 4,5-dimethyl-2-pentadecyl-	0.33±0.02[a]	n.d.	n.d.	n.d.
p-Cymene	2.05±0.04[a]	1.80±0.05[b]	1.07±0.05[c]	0.82±0.07[d]
Tetradecane	0.16±0.02[a]	0.111±0.010[ab]	0.10±0.02[bc]	0.06±0.04[c]
**TOTAL Others**	**10.5±0.8[a]**	**3.37±0.07[b]**	**2.12±0.10[c]**	**1.6±0.2[c]**

* The relative abundance of each volatile compound is expressed as a percentage of the total peak area in the chromatogram, after normalization to the peak area of the internal standard. Data are representative of three independent

experiments (n = 3) and are presented as mean ± SD. Values within a row with different superscript letters are significantly different ($p < 0.05$). n.d., not detected.

[1] Control carob (C0) and carob-wheat (CW0) sourdoughs were fermented for 24 h (_24) at 30°C, followed by six backslopping steps (R1 to R6) under the same conditions, except for the final refreshment, where fermentation was extended to 48 h (_48). Only data from control, R3, and R6 sourdoughs are shown. For more details, see the Materials and Methods section.
