# Supplementary material for: The Effects of Sourdough Fermentation on the Biochemical Properties, Aroma Profile and Leavening Capacity of Carob Flour
Source: Foods. 2025 May 9;14(10):1677. doi: 10.3390/foods14101677 (PMC12111545; doi:10.3390/foods14101677)
Supplement: Supplementary file 1 [file foods-14-01677-s001.zip › Table S2.pdf]

**Table S2. Main volatile organic compounds (VOCs) in carob-wheat sourdough***

| COMPOUND | Sourdough[1] | | | |
|---|---|---|---|---|
| | **CW0** | **CWR3_24** | **CWR6_24** | **CWR6_48** |
| **ACIDS** | | | | |
| Acetic acid | 0.49±0.05[a] | n.d. | n.d. | n.d. |
| Propanoic acid, 2-methyl-; | 8.9±0.9[a] | 4.3±0.2[b] | 4.70±0.10[b] | 3.9±0.2[b] |
| Butanoic acid | n.d. | n.d. | 0.61±0.06[a] | 0.33±0.02[b] |
| 4-Ketopimelic acid | 0.82±0.08[a] | n.d. | n.d. | n.d. |
| Hexanoic acid | 3.4±0.3[a] | 2.0±0.3[b] | 1.03±0.12[c] | 2.0±0.6[b] |
| Octanoic acid | n.d. | 0.32±0.12[a] | n.d. | n.d. |
| **TOTAL Acids** | **13.57±1.06[a]** | **6.5±0.2[b]** | **6.34±0.12[b]** | **6.3±0.7[b]** |
| **ESTERS** | | | | |
| Ethyl Acetate | n.d. | 1.54±0.07[b] | 2.7±1.2[ab] | 3.32±0.05[a] |
| Methyl isobutyrate | 2.5±0.4[a] | n.d. | n.d. | n.d. |
| Propanoic acid, 2-methyl-, ethyl ester | n.d. | 4.3±0.2[b] | 7.7±0.7[a] | 8.5±0.2[a] |
| Butanoic acid, ethyl ester | n.d. | 1.23±0.05[c] | 1.46±0.03[b] | 1.72±0.03[a] |
| 1-Butanol, 3-methyl-, acetate | n.d. | n.d. | 0.69±0.03[b] | 0.89±0.05[a] |
| Isobutyl isobutyrate | 2.02±0.08[a] | 0.75±0.06[b] | 1.0±0.2[b] | 0.97±0.07[b] |
| Hexanoic acid, methyl ester | 3.0±0.4[a] | 1.18±0.05[b] | 0.62±0.02[bc] | 0.56±0.13[c] |
| Hexanoic acid, ethyl ester | 1.8±0.3[d] | 28.5±0.5[c] | 33.9±1.0[b] | 36.4±0.8[a] |
| Propanoic acid, 2-methyl, 3-methylbutyl ester | 0.98±0.05[d] | 1.67±0.045[c] | 2.27±0.13[b] | 2.70±0.14[a] |
| Propanoic acid, 2-methyl-, 2-methylbutyl ester | 0.385±0.010[c] | 0.65±0.03[b] | 0.75±0.04[a] | 0.74±0.04[a] |
| Formic acid, octyl ester | n.d. | 2.20±0.10[a] | n.d. | 0.56±0.03[b] |
| Heptanoic acid, ethyl ester | n.d. | 1.35±0.04[a] | 0.47±0.06[c] | 0.72±0.04[b] |
| Hexyl iso-butirate | 0.39±0.02[a] | 0.28±0.05[b] | n.d. | n.d. |
| 4-Octenoic acid, ethyl ether | n.d. | 0.392±0.005[b] | 0.44±0.05[b] | 0.830±0.003[a] |
| Octanoic acid, ethyl ester | 0.28±0.03[d] | 3.49±0.07[c] | 5.2±0.2[b] | 7.9±0.3[a] |
| Isopentyl hexanoate | n.d. | 0.282±0.007[ab] | 0.26±0.04[b] | 0.335±0.012[a] |
| 2(1H)-Naphtalenone, 3,4,4a,5,6,7,8,8a-octahydro-5a-hydroxy-4aa,7,7-trimethyl acetate | 0.78±0.07[a] | 0.27±0.03[b] | 0.24±0.02[bc] | 0.16±0.02[c] |
| Nonanoic acid, ethyl ester | n.d. | 0.35±0.05[c] | 0.55±0.10[b] | 0.83±0.03[a] |
| Butanoic acid, 1-methyloctyl ester | 0.24±0.04[a] | n.d. | n.d. | n.d. |
| Ethanol, 2-(2-butoxyethoxy)-, acetate | 0.34±0.07[a] | n.d. | n.d. | n.d. |
| β-Phenylethyl butyrate | n.d. | n.d. | 0.37±0.03[a] | 0.15±0.10[b] |
| Hexanoic acid, 2-phenylethyl ester | n.d. | 0.24±0.03[a] | n.d. | n.d. |
| Ethyl 2-[(4-methylphenyl) amino] propanoate | 0.39±0.03[a] | n.d. | n.d. | n.d. |
| 2,2,4-trimethyl-1,3-pentadienol diisobutyrate | 7.6±0.3[a] | n.d. | n.d. | n.d. |
| **TOTAL Esters** | **20.7±1.0[d]** | **48.7±0.5[c]** | **58.6±0.6[b]** | **67.2±1.4[a]** |
| **ALDEHYDES and KETONES** | | | | |
| Butanal, 3-methyl- | 3.4±0.5[a] | n.d. | n.d. | n.d. |
| Butanal, 2-methyl- | 9.86±1.12[a] | n.d. | n.d. | n.d. |
| Hexanal | 2.5±0.5[a] | n.d. | n.d. | n.d. |
| Benzaldehyde | 0.59±0.02[a] | n.d. | n.d. | n.d. |
| Benzeneacetaldehyde | 0.45±0.09[a] | n.d. | n.d. | n.d. |
| 1H-Pyrrole 2-carboxialdehyde, 1-ethyl | 0.24±0.05[a] | n.d. | n.d. | n.d. |
| 2-Octenal | 0.52±0.09[a] | n.d. | n.d. | n.d. |
| Nonanal | 2.1±0.3[a] | 0.29±0.03[b] | 0.26±0.02[b] | n.d. |
| Ethanone, 1-(1H-pyrrol-2-yl)- | 3.0±0.8[a] | 2.36±0.12[a] | 1.09±0.10[b] | 0.85±0.12[b] |

**Table S2 (CONTINUATION)***

| | Sourdough[1] | | | |
|---|---|---|---|---|
| **COMPOUND** | **CW0** | **CWR3_24** | **CWR6_24** | **CWR6_48** |
| 2-Nonanone | 4.29±0.13$^{a}$ | 2.55±0.10$^{b}$ | 1.01±0.08$^{c}$ | n.d. |
| 3-Nonen-2-one | 0.29±0.03$^{a}$ | n.d. | n.d. | n.d. |
| 2-Undecanone | 1.6±0.2$^{a}$ | 0.20±0.02$^{b}$ | 0.20±0.02$^{b}$ | n.d. |
| 2-Tridecanone | 0.31±0.07$^{a}$ | 0.14±0.03$^{b}$ | 0.107±0.008$^{b}$ | n.d. |
| 3-Pentanedecanone | 0.48±0.04$^{a}$ | 0.076±0.006$^{b}$ | n.d. | n.d. |
| **TOTAL Aldehydes and Ketones** | **29.7±2.2$^{a}$** | **5.6±0.3$^{b}$** | **2.7±0.2$^{bc}$** | **0.85±0.12$^{c}$** |
| **ALCOHOLS** | | | | |
| 2-Methyl-1-propanol | n.d. | 1.29±0.06$^{c}$ | 1.74±0.05$^{a}$ | 1.49±0.09$^{b}$ |
| 3-Methyl butanol | 0.39±0.09$^{c}$ | 10.1±0.4$^{a}$ | 10.4±0.3$^{a}$ | 7.8±0.2$^{b}$ |
| 2-Methyl butanol | 0.34±0.08$^{d}$ | 9.4±0.3$^{b}$ | 10.0±0.4$^{a}$ | 7.10±0.06$^{c}$ |
| 1-Hexanol | 2.8±0.4$^{a}$ | 2.26±0.08$^{a}$ | 1.56±0.09$^{b}$ | 2.2±0.2$^{a}$ |
| 2-Heptanol | 0.73±0.07$^{b}$ | 0.51±0.03$^{c}$ | 1.38±0.03$^{a}$ | 1.27±0.02$^{a}$ |
| 1-Octen-3-ol | 0.37±0.03$^{a}$ | n.d. | n.d. | n.d. |
| 2-Ethyl-1-hexanol | 0.31±0.02$^{a}$ | n.d. | n.d. | n.d. |
| 2-Nonanol | 0.63±0.04$^{b}$ | 1.09±0.03$^{a}$ | 1.16±0.15$^{a}$ | 1.02±0.09$^{a}$ |
| Phenylethyl alcohol | n.d. | 8.9±0.3$^{a}$ | 3.5±0.3$^{b}$ | 3.3±0.261$^{b}$ |
| 1-Nonanol | 0.58±0.15$^{b}$ | 1.00±0.10$^{a}$ | 0.55±0.12$^{b}$ | n.d. |
| 1-Dodecanol | 0.47±0.12$^{a}$ | n.d. | n.d. | n.d. |
| **TOTAL Alcohols** | **6.6±0.6$^{d}$** | **34.5±0.6$^{a}$** | **30.3±0.7$^{b}$** | **24.2±0.6$^{c}$** |
| **FURANS** | | | | |
| 3(2H)-Furanone, dihydro-2-methyl | 0.68±0.08$^{a}$ | n.d. | n.d. | n.d. |
| Furfural | 13.5±0.3$^{a}$ | n.d. | n.d. | n.d. |
| 2-Furanmethanol | 0.89±0.09$^{a}$ | 0.58±0.03$^{b}$ | 0.5±0.2$^{b}$ | 0.46±0.04$^{b}$ |
| Ethanone, 1-(2-furanyl)- | 1.17±0.03$^{a}$ | 0.36±0.03$^{bc}$ | 0.40±0.05$^{b}$ | 0.28±0.04$^{c}$ |
| 2-Furancarboxaldehyde, 5-methyl- | 0.74±0.13$^{a}$ | n.d. | n.d. | n.d. |
| 2-pentylfuran | 0.33±0.02$^{a}$ | n.d. | n.d. | n.d. |
| Dihydro-3-methylene-5-methyl-2-furanone | 3.2±0.6$^{a}$ | n.d. | n.d. | n.d. |
| Butanoic acid, 2-furanylmethyl ester | 1.24±0.02$^{b}$ | 1.70±0.04$^{a}$ | 0.50±0.02$^{c}$ | 0.322±0.010$^{d}$ |
| 7-Benzofuranamine, 2,3-dihydro-2,2-dimethyl | 0.27±0.03$^{a}$ | 0.13±0.02$^{b}$ | n.d. | n.d. |
| **TOTAL Furans** | **22.0±0.6$^{a}$** | **2.76±0.09$^{b}$** | **1.4±0.3$^{c}$** | **1.06±0.07$^{c}$** |
| **OTHERS** | | | | |
| Propane, 2-ethoxy-2-methyl- | 0.39±0.07$^{a}$ | n.d. | n.d. | n.d. |
| Silanediol, dimethyl- | 3.4±0.3$^{a}$ | 1.05±0.09$^{b}$ | n.d. | n.d. |
| Dimethyl sulfide | 1.31±0.12$^{a}$ | n.d. | n.d. | n.d. |
| 1,3-Dioxolane, 4,5-dimethyl-2-pentadecyl | 0.30±0.02$^{a}$ | n.d. | n.d. | n.d. |
| o-Cymene | 1.8±0.2$^{a}$ | 0.75±0.06$^{b}$ | 0.57±0.06$^{bc}$ | 0.438±0.009$^{c}$ |
| Tetradecane | 0.217±0.002$^{a}$ | 0.09±0.03$^{b}$ | 0.10±0.05$^{b}$ | n.d. |
| **TOTAL Others** | **7.430±0.274$^{a}$** | **1.89±0.10$^{b}$** | **0.68±0.12$^{c}$** | **0.438±0.009$^{c}$** |

* The relative abundance of each volatile compound is expressed as a percentage of the total peak area in the chromatogram, after normalization to the peak area of the internal standard. Data are representative of three independent experiments (n = 3) and are presented as mean ± SD. Values within a row with different superscript letters are significantly different ($p < 0.05$). n.d., not detected.

[1] Control carob (C0) and carob-wheat (CW0) sourdoughs were fermented for 24 h (_24) at 30°C, followed by six backslopping steps (R1 to R6) under the same conditions, except for the final refreshment, where fermentation was

extended to 48 h (_48). Only data from control, R3, and R6 sourdoughs are shown. For more details, see the Materials and Methods section.
